# Prevalence of Trachoma from 66 Impact Surveys in 52 Woredas of Southern Nations, Nationalities and Peoples’ and Sidama Regions of Ethiopia, 2017–2019

**DOI:** 10.1080/09286586.2022.2065313

**Published:** 2022-04-27

**Authors:** Dawit Seyum, Nigusie Fetene, Tezera Kifle, Habtamu Negash, Temesgen Kabeto, Mulatu Gebre, Tadesse Data, Tafese Tadele, Getahun Abayo, Asfaw Wondimu, Robert Butcher, Ana Bakhtiari, Rebecca Willis, Sarah Boyd, Cristina Jimenez, Nebiyu Negussu, Fentahun Tadesse, Fikreab Kebede, Michael Dejene, Anthony W. Solomon, Emma M. Harding-Esch, Alemayehu Sisay

**Affiliations:** a Orbis International Ethiopia, Orbis International Ethiopia, Addis Ababa, Ethiopia; b Southern Nations, Nationalities and Peoples’ Regional Health Bureau, Hawassa, Ethiopia; c Durame Hospital, Durame, Ethiopia; d Eyen Consultancy, Addis Ababa, Ethiopia; e Sightsavers, Haywards Heath, UK; f ITI, Task Force for Global Health, Decatur, Georgia, USA; g Ministry of Health, Federal Ministry of Health, Addis Ababa, Ethiopia; h Sightsavers, Addis Ababa, Ethiopia; i Department of Control of Neglected Tropical Diseases, World Health Organization, Geneva, Switzerland; j Clinical Research Department, London School of Hygiene & Tropical Medicine, London, UK

**Keywords:** Ethiopia, trachoma, trichiasis, Southern Nations, Nationalities and Peoples’, Region, Sidama Region

## Abstract

**Purpose:**

Trachoma is endemic in Southern Nations, Nationalities and Peoples’ (SNNP) and Sidama regions of Ethiopia. We aimed to measure the prevalence of trachomatous inflammation—follicular (TF) among children aged 1–9 years and the prevalence of trachomatous trichiasis (TT) unknown to the health system among people aged ≥15 years following interventions for trachoma in 52 woredas of SNNP and Sidama regions.

**Methods:**

From 2017 – 2019, 66 two-stage cluster sampling cross-sectional population-based surveys were carried out in 52 woredas (third-level administrative divisions) using a standardized World Health Organization-recommended survey methodology. This included one impact survey in 40 woredas, two consecutive impact surveys in 10 woredas and three consecutive impact surveys in two woredas. Water, sanitation and Hygiene (WASH) access was assessed using a modified version of the United Nations Children’s Fund/WHO Joint Monitoring Programme questionnaire.

**Results:**

By the end of this survey series, 15 (23%) of the woredas had met the active trachoma elimination threshold (TF prevalence <5%) and 12 (18%) had met the TT threshold (TT ≤ 0.2%). Regarding WASH coverage, 20% of households had access to an improved drinking water source within a 30-min journey and 3% had an improved latrine. There was strong evidence that TF was less common in 4 – 6-year-olds and 7 – 9-year-olds than 1 – 3-year-olds.

**Conclusion:**

Based on the findings, further antibiotic mass drug administration is required in 37 woredas and active TT case finding is needed in 40 woredas. In these surveys, access to WASH facilities was very low; WASH improvements are required.

## Introduction

Of the estimated 2.2 billion people around the world who are visually impaired, at least one billion are suffering from avoidable causes of visual impairment.^1^ Blindness and low vision are major public health problems in Ethiopia. Females and residents of rural areas are at greater risk of eye problems.^2^

Trachoma, caused by *Chlamydia trachomatis*, is the leading infectious cause of blindness.^3^ It is responsible for blindness or visual impairment of about 2 million people worldwide.^4^ In May 2020, trachoma was a public health problem in 44 countries, with 137 million people estimated to be at risk of trachoma-related blindness.^4^ Trachoma remains a major cause of avoidable blindness among populations in areas in which poverty, over-crowding, and poor personal and environmental hygiene increase risk of its transmission.^5,6^

Infection is transmitted through transfer of infected ocular or nasal secretions from person to person through eye-seeking flies, fingers, and fomites.^7–9^ Repeated episodes^10^ of infection and inflammation can cause scarring of the inner part of the eyelids. In some individuals, trachomatous trichiasis (TT), in which one or more eyelashes from the upper eyelid^11,12^ touch the eyeball, ensues. TT is an extremely painful eye condition.^13^ It can be corrected surgically but, if left untreated, may lead to corneal opacification, low vision, and blindness.

The World Health Organization (WHO) leads an international alliance for the global elimination of trachoma. The Alliance is guided by the WHO-endorsed SAFE strategy; the **S** component of the strategy is lid surgery to correct TT; **A** is antibiotics to clear infection, delivered as annual rounds of mass drug administration (MDA) to populations in which the TF prevalence among 1 − 9-year-olds is ≥5%; **F** is promotion of facial cleanliness; and **E** is environmental improvement.^14^

The criteria for elimination of trachoma as a public health problem are: (i) prevalence of TT unknown to the health system <0.2% among people aged ≥15 years, and (ii) prevalence of TF < 5% among children aged 1–9 years, in each endemic district, plus (iii) evidence that the health system can continue to identify and manage incident cases of TT.^15^

In Southern Nations, Nationalities and Peoples’ Region (SNNPR) of Ethiopia, baseline population-based trachoma surveys were conducted from February 2013 to May 2014 in collaboration with the Global Trachoma Mapping Project (GTMP),^16^ covering a total of 40 evaluation units (EUs) incorporating 113 woredas. (Woredas are third-level administrative divisions equivalent to districts, positioned after regional states and zones. EUs are the normal administrative unit for health-care management, consisting of a population unit between 100,000 and 250,000 persons.) The surveys showed that the age- and gender-adjusted EU-level prevalence of TT ranged from 0.0% in Hula woreda to 6.1% in Cheha woreda. The age-adjusted EU-level prevalence of TF ranged from 2.3% in Geta and Gumer woredas to 48.5% in Amaro and Burji woredas.^16^ These baseline surveys included both SNNP and Sidama regions: prior to 2020, Sidama region was part of SNNPR.

The SAFE strategy was implemented at scale across SNNPR (and Sidama) from 2013 and is ongoing at the time of publication. The number of rounds of MDA delivered in each woreda was guided by WHO recommendations,^14^ meaning woredas received between one and five rounds of MDA (depending on baseline prevalence) before the first impact survey was conducted. In most cases, MDA was delivered annually; in two woredas (Mareko, TF prevalence in 1 − 9-year-olds 36% and Siliti, TF prevalence 27%), their 2017 MDA rounds were missed and their schedule was resumed in 2018. Both woredas still received the recommended number of rounds despite the gap. By the end of the study period, the majority of woredas had received three annual rounds of MDA. Those with the highest TF prevalence had received up to seven rounds. All delivered MDA rounds achieved a programmatically estimated coverage >80%. Coverage was supervised at the kebele (the smallest administrative unit) level. Coverage supervision started on the fifth day of the distribution and those who reported not taking antibiotic were offered treatment in a ‘mop up’ programme. This contributed to the high woreda-level MDA coverage. From 2015 to 2019, >70,000 TT surgeries were performed in surveyed woredas, equating to approximately 14,000 per year. Facial cleanliness promotion and environmental improvement activities were carried out in surveyed districts. These activities included community and school-based awareness raising activities, latrine construction, eye health education at health facilities and in schools, and distribution of health education materials.

WHO recommends impact surveys 6–12 months after the last planned MDA round. If the impact survey TF prevalence in 1–9-year-olds is ≥5%, annual MDA should be continued with the number of rounds determined by prevalence category, followed by another impact survey.^17^ The aim of this series of surveys was to measure the prevalence of TF among children aged 1–9 years and the prevalence of TT unknown to the health system among people aged ≥15 years following interventions for trachoma.

## Materials and methods

### Study design and setting

From 2017 to 2019, community-based cross-sectional surveys were employed in 52 woredas of SNNP and Sidama regions. Throughout this survey series, each woreda was considered an EU. These woredas were chosen as they had completed the required number of rounds of MDA based on their baseline TF prevalence, following WHO guidance. Over the course of this series, one trachoma impact survey (TIS) was conducted in 40 woredas, two TISs were conducted in 10 woredas and three TISs in two woredas. Sixty-six surveys were conducted in total. Standard quality-assurance and quality-control measures for trachoma prevalence surveys were implemented.^18,19^

### Training of graders and recorders

Internationally standardized five-day initial and refresher Tropical Data training sessions were run immediately before each series of surveys.^20^ Grader training was focused on examination of eyes for signs of trachoma using WHO’s simplified trachoma grading system.^21^ Only those who passed the slide test with an inter-grader agreement kappa^22^ of ≥0.7 took the field reliability test, in which grader trainees graded 50 eyes of 50 children; there they needed a kappa for TF of ≥0.7, compared to a certified Tropical Data grader trainer.^20^ Graders were trained to look for the presence or absence of other clinical signs (TT, TS, and TI) but were not tested on diagnostic accuracy of these signs. Similarly, recorder trainees underwent intensive training on the survey tools, recognition of water, sanisanitation, hygiene (WASH) facilities and data recording using the Tropical Data app in Android phones. They were deployed for surveys if they scored ≥90% in the recorder reliability test.

Graders and recorders who had previously taken part in GTMP and/or Tropical Data surveys were preferentially selected as team members for subsequent surveys. New trainees participated in the initial training programme before starting survey work.^20^

Graders and recorders who had not attended an initial or refresher training within the last 12 months attended a refresher training course before starting work. Teams were supervised by field supervisors; one field supervisor was responsible for the supervision of 6–7 teams.

### Sample size determination and sampling procedure

The required sample size for the survey was determined using the single population proportion for precision formula. The design effect was set at 2.63 and an inflation factor of 1.2 was applied to account for non-response. Based on those assumptions, a sample of 1,164 children aged 1–9 years would be needed to measure a prevalence of TF of 4% with a precision of ±2% at the 95% confidence level.^23^

The number of clusters needed from each woreda was determined by dividing the total targeted number of children aged 1–9 years by the mean number of house-holds a team could comfortably survey per day (30) multiplied by the mean number of 1–9-year-olds per household (1.5 in Ethiopia, according to census figures.^24^) Therefore, in each woreda, 26 clusters each with 30 households were required. A two-stage cluster sampling technique was used.

In the first stage of sampling, 26 kebeles were randomly selected from the list of kebeles available in each woreda. In the second stage of the sample selection, compact segment sampling was employed to pick 30 households from the selected kebele.

### Data collection methods and tools

Data were captured electronically using a purpose-built Secure Data Kit-based Android smartphone application. Once household heads agreed for their household to be enrolled, Global Positioning System (GPS) coordinates of the household were recorded and a WASH questionnaire, modified for trachoma surveys from the United Nations Children’s Fund/WHO Joint Monitoring Programme household questionnaire,^19,25^ was administered to the head of the household or their nominee.

Consenting individuals were examined by the graders for signs of TT, TF, and TI according to the WHO simplified system.^21^ Graders used 2.5× binocular loupes for magnification, sunlight, or a torch for illumination, and from 2019 onwards, follicle size guides^26^ to assist with the diagnosis of TF.

### Data analysis

Data collected in the field were uploaded to Tropical Data servers in near-real time, dependent on internet connectivity. Data were checked and cleaned by the Tropical Data data manager. Cases of TT who reported not having been offered surgery or who could not remember being offered surgery were classified as “unknown to the health system”. The mean of the age-adjusted cluster-level TF proportions was taken as the woreda-level prevalence of TF among 1 − 9-year-old children and the mean of the age- and gender-adjusted cluster-level TT proportions was taken as the woreda-level prevalence of TT among people aged ≥15 years. WASH facilities were categorized according to UNICEF/WHO JMP definitions.^27^

The association between TF and individual- and household-level variables was assessed using mixed-effects binomial logistic regression analyses, following methods described previously.^16^ The relationship between TT, age, and gender was assessed in the same manner. For models where TT was the dependent variable, management status of TT was felt to be affected by factors beyond those measured in the surveys and was therefore not specified in the models. Additionally, the development of TT is thought to be a cumulative result of a lifetime of stimuli,^28^ which the WASH infrastructure of a household at a single, recent timepoint was considered not to fully capture. Association between TT and WASH variables was therefore not tested.

For all models, woreda, cluster and household of residence were considered for random-effects variables. The random-effects variables were selected using step-wise subtraction of variables from a null model containing all three levels. Once the random-effects parameters were selected, correlation was assessed between all variables and colinear variables were omitted. Univariable associations were then tested using likelihood ratio tests to compare univariable to null models. Multivariable models were assembled from variables that were considered significant (p <.05) in univariable analysis. Fixed effects coefficients were exponentiated to achieve odds ratios (ORs), and confidence intervals (CIs) around ORs were calculated using Wald’s method.

### Ethical considerations

Prior to the commencement of the surveys, ethical clearance was obtained from SNNPR Health Bureau ethical clearance committee (PMG-19/11741). The London School of Hygiene & Tropical Medicine Observational Ethics Committee (16105) approved Tropical Data survey support.

All residents aged ≥1 year in selected households were eligible to take part in the study. Before participating, enrollees were informed about the study objectives and asked to give their verbal consent to take part. A parent/guardian gave verbal consent for children aged <18 years. In the absence of a parent/guardian, another adult member of the household gave verbal consent on behalf of children. Consent was recorded in the Android-based data collection form.

Participants identified with active trachoma were treated with 1% tetracycline eye ointment. Participants found to have TT or other eye conditions were referred to Primary Eye Care Units for treatment.

## Results

### Description of study population

A total of 221,770 people aged ≥1 year were enumerated in the 66 woreda-level surveys. This included 52 first TIS, 12 second TISs and two-third TISs. Overall, 201,561 (91%) of the enumerated individuals were present at home at the time of visit and consented for examination.

The mean number of people examined from each woreda was 3,054 and 55% of examined participants were female (Table 1).

### Prevalence of TF in 1 − 9-year-olds

**Figure 1:**
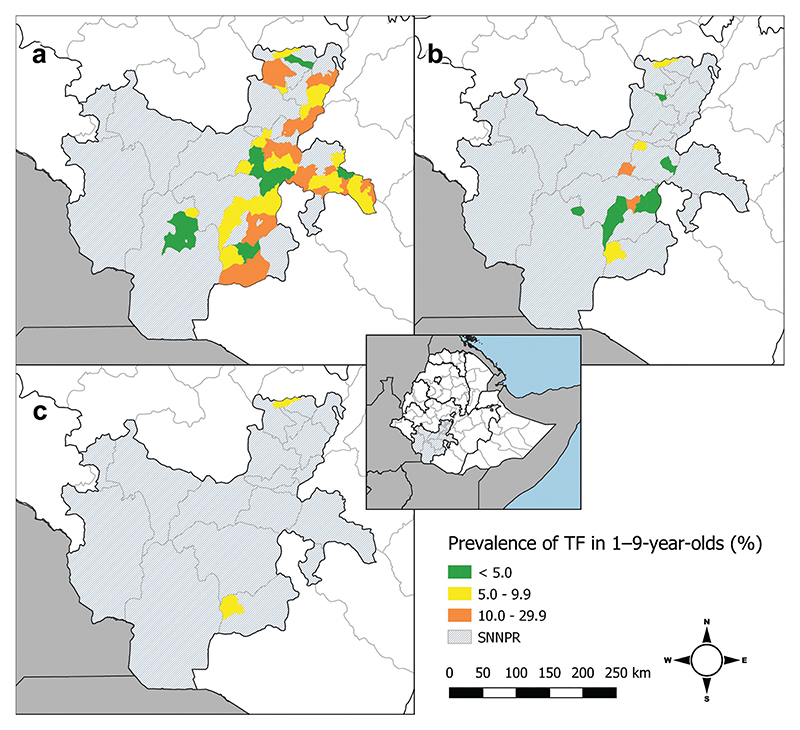
Prevalence of TF among children aged 1 − 9 years in trachoma impact surveys in SNNP and Sidama regions (SNNPR), Ethiopia, during the (A) first, (B) second and (C) third impact survey during this series. The boundaries and names shown, and the designations used on this map do not imply the expression of any opinion whatsoever on the part of the authors, or the institutions with which they are affiliated, concerning the legal status of any country, territory, city or area or of its authorities, or concerning the delimitation of its frontiers or boundaries.

A total of 68,037 children aged 1–9 years were examined in the 66 surveys. The age-adjusted woreda-level prevalence of TF among children aged 1 − 9 years ranged from 1.95% (95% CI: 0.71 − 3.69) in Mihur Aklil woreda to 25.43% (95% CI: 19.90 − 30.43) in Damot Pullassa woreda. Of the total 66 surveys, the prevalence of TF was <5% in 15 (23%) and ≥10% in 21 (32%) (supplementary Table 1, Figure 1A-1C). No woreda had a TF prevalence ≥30%.

The surveys were conducted from 2017 to 2019. Forty woredas had only one survey and 12 woredas had more than one survey during that period (Figure 1; supplementary Table 1).

In the first round of TISs (n = 52), 52,841 children aged 1 − 9 years were examined and the age-adjusted woreda-level prevalence of TF among children aged 1 − 9 years ranged from 1.95% (95% CI 0.71–3.69) in Mihur Aklil woreda to 25.43% (95% CI 19.90 − 30.43) in Damot Pullassa woreda. The prevalence of TF was <5% in eight woredas, between 5.0 − 9.9% in 25 woredas and between 10.0 − 29.9% in 19 woredas (Figure 1 A).

In the second round of TISs (n = 12), 13,113 children aged 1 − 9 years were examined and the age-adjusted woreda-level prevalence of TF among children aged 1 − 9 years ranged from 2.62% (95% CI 1.34 − 4.22) in Chencha woreda to 11.36% (95% CI 7.84 − 16.03) in Kindo Didaye woreda. The prevalence of TF was <5% in seven woredas, between 5.0 − 9.9% in three woredas and ≥10% in two woredas (Figure 1B).

In the third round of TISs (n = 2), 2,083 children aged 1 − 9 years were examined and the adjusted prevalence of TF was between 5.0 − 9.9% in both woredas (Figure 1C).

### Prevalence of TT among adults

**Figure 2:**
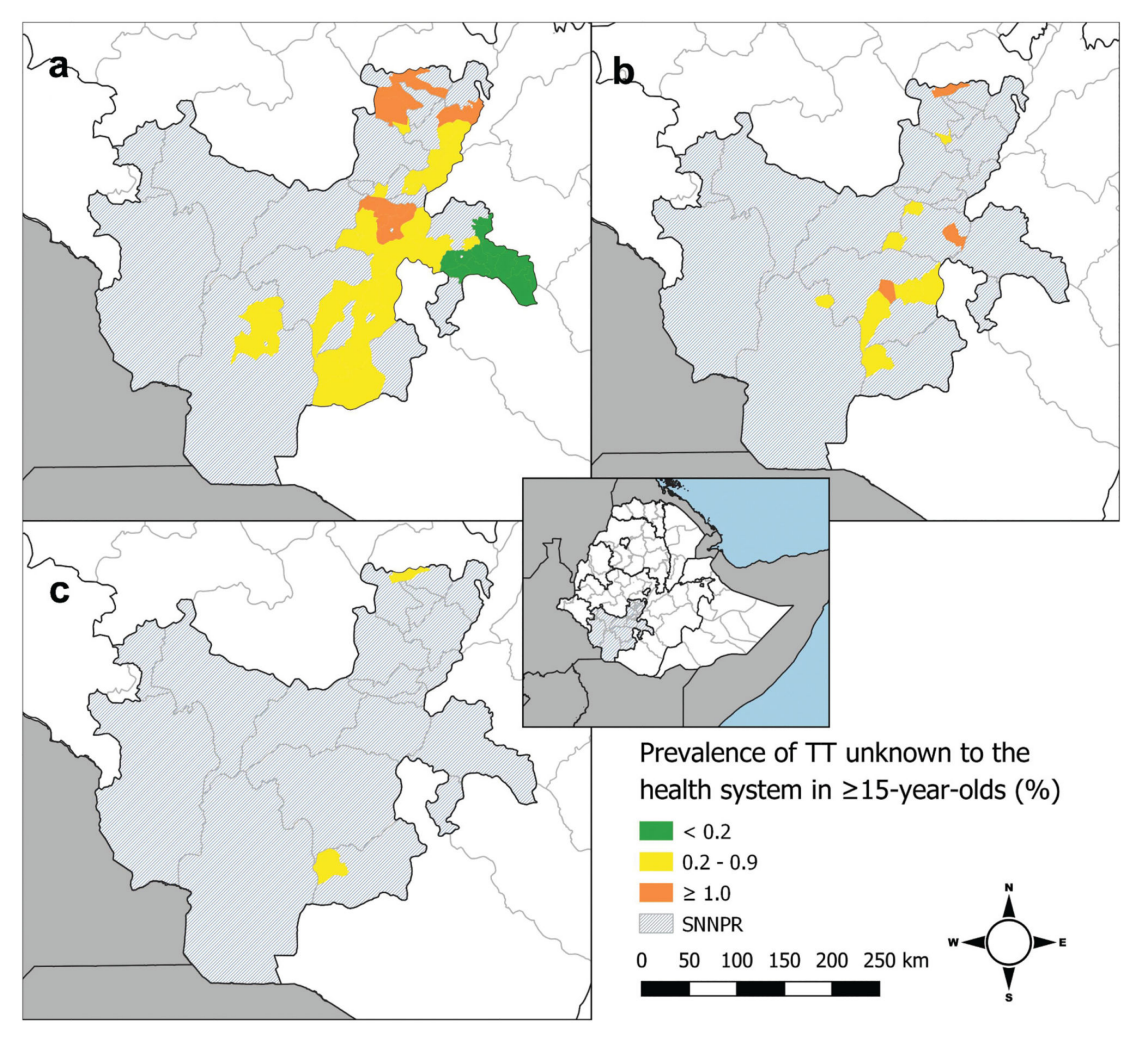
Prevalence of TT unknown to the health system in people aged ≥15 years and above in SNNP and Sidama regions, Ethiopia, during the (A) first, (B) second and (C) third impact survey during this series. The boundaries and names shown, and the designations used on this map do not imply the expression of any opinion whatsoever on the part of the authors, or the institutions with which they are affiliated, concerning the legal status of any country, territory, city or area or of its authorities, or concerning the delimitation of its frontiers or boundaries.

A total of 108,293 adults aged ≥15 years were examined. The age- and gender-adjusted prevalence of TT unknown to the health system in ≥15 years old ranged from 0.00% (95% CI not calculated) in Bursa and Hula woredas to 2.40% (95% CI 1.72 − 3.17%) in Kebena woreda. The adjusted prevalence of TT unknown to the health system in those aged ≥15 years was <0.2% in 12 woredas (supplementary Table 2, Figure 2).

There were 85,818 adults examined in the 52 woredas receiving their first TIS. The adjusted pre-valence of TT unknown to the health system in ≥15-year-olds ranged from 0% in Bursa woreda to 2.40% (95% CI: 1.72–3.17) in Kebena woreda. The adjusted prevalence of TT unknown to the health system in those aged ≥15 years was between <0.2% in 12 woredas, and ≥0.2% in the other 40 woredas (Figure 2A).

A total of 19,340 adults aged ≥15 years were examined in the second round of TISs (n = 12). In these surveys, the adjusted prevalence of TT unknown to the health system in those aged ≥15 years was ≥0.2% and 0.9% in all 12 woredas (Figure 2 B). During the third-round TISs (n = 2), 3,135 adults were examined and the adjusted prevalence of TT unknown to the health system in those aged ≥15 years was ≥0.2% in both woredas (Figure 2 C).

### Change in TF prevalence over time

**Figure 3:**
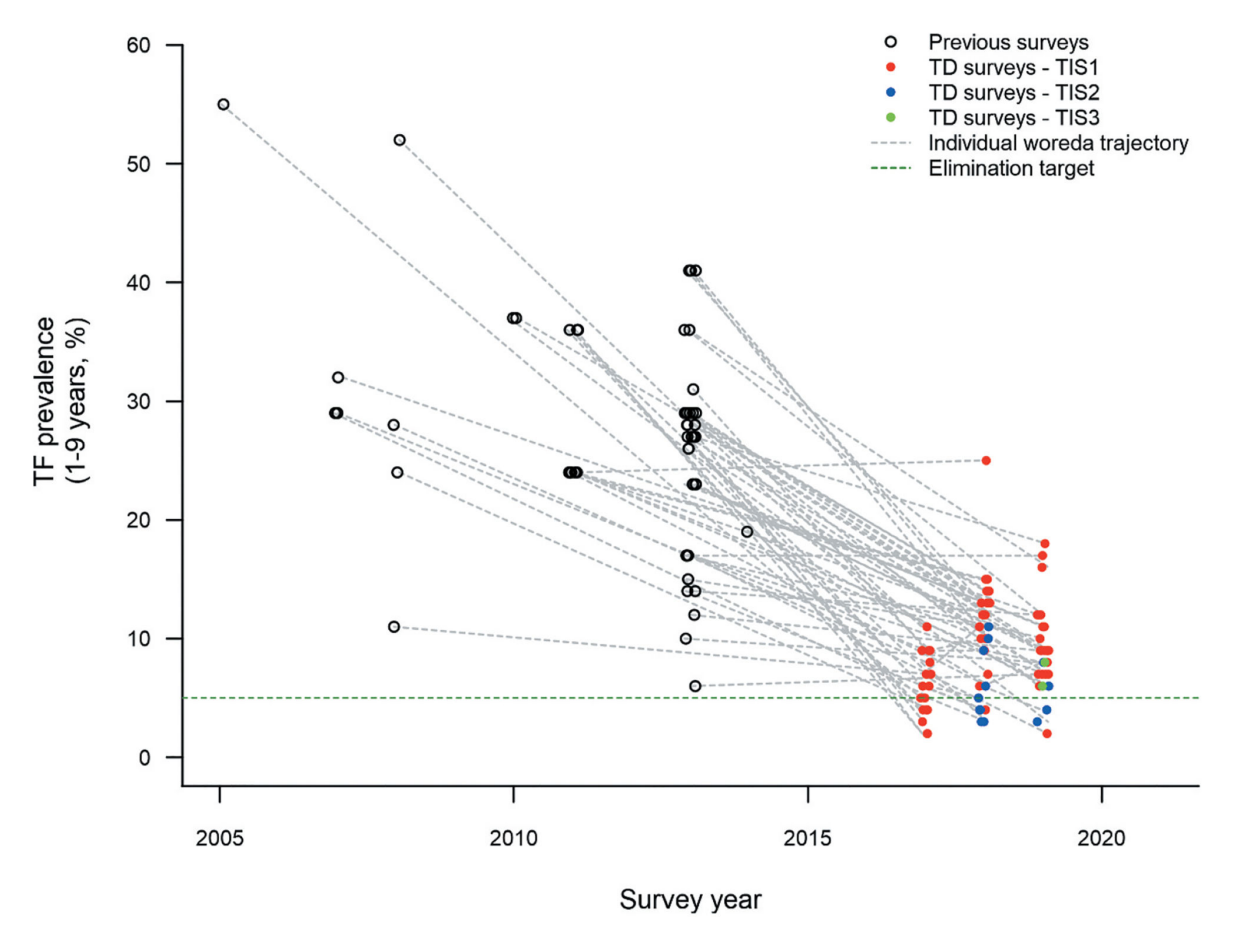
Change in trachomatous inflammation—follicular (TF) prevalence in children aged 1 – 9 years in populations of SNNP and Sidama regions, Ethiopia, which had had multiple estimates of this index by 2019. Prevalence estimates from 2017 – 2019 are trachoma impact survey data generated with support from Tropical Data (TD) and presented in this manuscript. Prevalence estimates from 2013 were generated as part of the Global Trachoma Mapping Project.^16^ Pre-2013 estimates were generated from Orbis baseline trachoma prevalence survey (unpublished). Baseline surveys were carried out using different EU boundaries to those used in these impact surveys, and therefore direct comparisons are not appropriate.

Generally, there was a declining TF prevalence among children aged 1–9 years in all woredas that received MDA. The elimination threshold of TF < 5% in children aged 1 – 9 years was achieved in 15 woredas by 2019 (Figure 3), although only nine woredas reached the threshold after the first TIS.

### Household-level WASH Access

A total of 53,020 households were visited and 20% of households had access to improved drinking water within a 30-min journey. Only 3% households had an improved latrine, and 2% households had a latrine with a handwashing station with water and soap (supplementary Table 3).

### Associations of TF

Following stepwise model assembly, household and cluster of residence were included as random-effects variables to account for disease clustering at those levels, for both TF and TT association models. Household-level water or sanitation access could not be categorized in the households of 19 children, and these children were excluded from the analysis. The regression analysis was therefore conducted on 68,018 children. There was strong evidence that TF was more common in younger children than older children, and there was a strong association between having TF and living in a household located further from a washing water source, not having a latrine, practising open defecation or accommodating a higher number of 1–9-year-olds.

There was strong evidence that TT was more common in women (aOR compared to men: 3.15, 95% CI: 2.84 − 3.50) and in older people. Compared to 15 − 34-year-olds, the aOR for TT was 3.79 (95% CI: 3.37 − 4.3) in 35 − 55-year-olds, 9.09 (95% CI: 8.03 − 10.29) in 55 − 75-year-olds, and 11.47 (95% CI: 9.00 − 14.60) in ≥75-year-olds (Table 2).

## Discussion

Progress has been made against trachoma in this part of Ethiopia, though we are still some distance from the finish line. The reduction of TF prevalence in those woredas that had repetitive TISs was encouraging. In 54 of 66 woreda-level surveys, however, the prevalence of TT unknown to the health system among adults aged ≥15 years was above the WHO elimination threshold level. About 20% of the households had access to improved drinking water within a 30-min return journey. However, only 3% and 2% of households had an improved latrine and a latrine with a handwash station with water and soap, respectively. Generally, WASH coverage was poor both in SNNP and Sidama regions.

There was strong evidence for TF being more common in younger people and TT being more common in older people. This is consistent with trachoma’s usual epidemiology.^29^ TF was equally distributed between male and female children, but women had odds of TT that were three times greater than that for men. This closely reflects findings elsewhere on the age and gender distribution of trachoma.^30,31^ There was strong evidence that poor access to WASH facilities was associated with having TF. The findings are generally consistent with findings at baseline in these regions, where TF was associated with living in a household using surface water as a primary washing water source and with living in a household with unimproved or no latrine.^16^ This is a good demonstration that people with TF, typically, live in the more deprived sectors of society and emphasizes the need for the F and E components of SAFE. Given the scale of the surveys here, we considered whether the dataset could be used to identify variables at community level associated with programmatic outcomes, such as achieving a prevalence of TF < 5% after MDA. As surveys are powered at EU level in order to make EU-level programmatic decision, community (cluster)-level analyses of association may not be programmatically informative and amalgamating to woreda level would reduce the sample size within this dataset to the point that complex models would be underpowered. Large-scale analyses combining survey datasets from multiple countries would be needed to analyse geospatial and program delivery-level outcomes; such analyses are already in progress.^32^

There are some limitations to this study. In Figure 3, new data are compared to baseline trachoma survey data, purportedly to illustrate the change in TF over time. However, baseline surveys were carried out using different EU boundaries, and therefore direct comparisons are subject to error. This limits our ability to truly understand the change in TF. It does not impede successful completion of the aim of this survey series, which was to identify where interventions are needed in the future. Another limitation is the sample size used to estimate TT prevalence. We acknowledge that these surveys were primarily powered to estimate prevalence of TF in children. The scarcity of TT compared to TF means the surveys were slightly under-powered to accurately estimate TT prevalence. Modeling data used to inform TT survey design suggests 2818 adults or 30 clusters are needed to estimate TT prevalence with adequate precision.^33^

Based on WHO recommendations, at least three more rounds of annual antibiotic MDA are needed in 21 woredas that had a TF prevalence ≥10%. One more round of MDA is required in 16 other woredas before further impact surveys. In addition, the F and E components of the SAFE strategy should be implemented across these regions. Fifteen woredas have now met the WHO elimination target for TF so no more rounds of antibiotic MDA are needed, but the F and E components of the SAFE strategy should continue; in these woredas, trachoma surveillance surveys are needed two years after the impact surveys took place to check for recrudescence.^14,17^

It is notable that the expected reduction of TF prevalence following the recommended number of rounds of MDA was not observed in a substantial proportion of the woredas surveyed. This is not unique to SNNP and Sidama.^34–36^ It presents a challenge to trachoma’s elimination as a public health problem. The reasons for this are not clear. There may be a number of environmental (e.g., low WASH access coverage), programmatic (e.g., insufficient MDA coverage) or biological factors that may cause persistence of TF at the woreda-level after MDA; operational research to better understand these factors in SNNP and Sidama should be a programmatic priority.

The prevalence of TT unknown to the health system among adults aged ≥15 years in SNNP and Sidama regions was above the WHO elimination target at the most recent survey in 40 woredas. Based on this, active TT case finding is recommended to meet the WHO elimination targets for TT.^14,37^

In conclusion, although there has been good progress against TF and TT in the surveyed woredas, trachoma remains an important public health problem in SNNP and Sidama regions. Continuing to implement the SAFE strategy, conducting operational research, relationships with partners, political commitment and funding opportunities will all be critical to eventual success.

## Figures and Tables

**Table 1:** Population aged ≥1 year participating in 66 Trachoma Impact Surveys in SNNP and Sidama regions, Ethiopia, 2017–2019.

Zone	Evaluation units	Enumerated	Absent	Refused	Other	Examined	Examined female (%)
Gamo	Boreda, Chencha, Dita, Kemba, Mirab Abaya, Arba Minch zuria, Dermalo	40,699	3,825	54	2	36,818	20,099 (55%)
Gurage	Cheha, Kebena, Mihur Aklil, Enemorna Ener, Endegagn, Mareko	29,068	2,298	36	2	26,732	14,621 (55%)
South Omo	North Aari, South Aari	9,097	563	9	0	8,525	4,536 (53%)
Wolayita	Humbo, Kindo Didaye, Kindo Koysha, Offa, Boloso Bombe, Boloso Sore, Damot Gale, Damot Pullasa, Damot Sore, Damot Woyde, Duguna Fango, Soddo zuria	50,958	4,808	97	4	46,049	25,389 (55%)
Kembata- Tembaro	Damboya, Kedida Gamela, Hadero Tunto zuria	10,773	1,064	19	0	9,690	5,392 (56%)
Sidama region	Aleta Chuko, Aleta Wondo, Arbegona, Aroresa, Bensa, Bona zuria, Bursa, Chire, Dara, Gorche, Loka Abaya, Hula, Wonsho, Malega	47,170	4,291	108	0	42,771	23,253 (54%)
Siltie	Dalocha, Lanforo, Sankura, Silti	13,397	827	11	1	12,558	6,863 (55%)
Halaba	Halaba Rural	3,728	367	4	0	3,357	1,857 (55%)
Konso	Konso	3,418	353	2	0	3,063	1,739 (57%)
Special woreda	Alle, Derashe	13,462	1,429	32	3	11,998	6,831 (57%)

**Table 2:** Association between trachomatous inflammation—follicular (TF) and individual- and household-level variables in 1 – 9-year-olds in trachoma impact surveys in Southern Nations, Nationalities and Peoples’ and Sidama regions of Ethiopia, 2017 – 2019. Household and cluster of residence were included as random-effects variables.

				Univariable analysis		Multivariable analysis	
Variable	Levels	No TF	TF	OR (95% CI)	P	aOR (95% CI)	^p^
Age group (years)	1 – 3	15,638	2,973	Ref	<0.001	Ref	<0.001
	4 – 6	22,117	2,362	0.45 (0.42 – 0.48)		0.46 (0.43 – 0.49)	
	7 – 9	24,189	739	0.10 (0.09 – 0.12)		0.10 (0.09 – 0.12)	
Gender	Male	31,289	3,125	Ref	0.0369	Ref	0.161
	Female	30,655	2,949	0.93 (0.88 – 1.00)		0.96 (0.90 – 1.03)	
Washing water source	Improved	35,841	3,549	Ref	0.205	Not tested	
	Unimproved	14,948	1,385	0.98 (0.88 – 1.09)			
	Surface	11,155	1,140	0.91 (0.82 – 1.01)			
Return journey to washing water source	≤30 minutes	24,019	2,071	Ref	<0.001	Ref	<0.001
	>30 minutes	37,925	4,003	1.23 (1.13 – 1.33)		1.25 (1.14 – 1.37)	
Latrine ownership	Private	45,878	4,211	Ref	<0.001	Ref	<0.001
	Shared	6,124	695	1.22 (1.09 – 1.37)		1.16 (1.02 – 1.31)	
	Open	9,942	1,168	1.36 (1.23 – 1.50)		0.85 (0.71 – 1.02)	
Latrine status	Improved	1,587	85	Ref	<0.001	Ref	<0.001
	Unimproved	12,884	1,606	2.12 (1.59 – 2.82)		1.59 (1.17 – 2.14)	
	Open	47,473	4,383	1.49 (1.13 – 1.97)		2.60 (1.85 – 3.66)	
Number of 1 – 9-year-olds in the household	1 – 2	34,777	3,212	Ref	<0.001	Ref	<0.001
	3 – 4	25,436	2,677	1.20 (1.11 – 1.28)		1.25 (1.15 – 1.35)	
	≥5	1,731	185	1.25 (1.00 – 1.58)		1.43 (1.11 – 1.85)	

aOR: adjusted odds ratio; CI: confidence interval; OR: odds ratio
